# Gut bacteria induce heterologous immune priming in *Rhodnius prolixus* encompassing both humoral and cellular immune responses

**DOI:** 10.1371/journal.ppat.1012947

**Published:** 2025-12-01

**Authors:** Carissa A. Gilliland, Loretta Mugo-Kamiri, Sara Martin, Kevin J. Vogel

**Affiliations:** 1 Entomology Department, The University of Georgia, Athens, Georgia, United States of America; 2 Department of Genetics, The University of Georgia, Athens, Georgia, United States of America; The University of Edinburgh, UNITED KINGDOM OF GREAT BRITAIN AND NORTHERN IRELAND

## Abstract

Insects lack the adaptive, antibody mediated responses of vertebrates, yet they possess a robust innate immune system capable of defending themselves against pathogens. Immune priming has been observed in multiple insect species, wherein exposure to a pathogen provides protection against subsequent infections by the pathogen. Heterologous immune priming has also been described, where presence of one bacterial species provides protection against another. We determined that *Rhodococcus rhodnii*, a gut symbiont of the kissing bug *Rhodnius prolixus,* induces strong heterologous immune priming, while axenic bugs lacking gut bacteria are highly susceptible to pathogens. Commensal *Escherichia coli* provides less robust protection. *R. rhodnii* must be alive within the insect as dead bacteria do not stimulate immune priming and pathogen resistance. Removal of *R. rhodnii* from the gut reduces resistance to pathogens while restoring it to axenic bugs improves pathogen resistance, though not completely. Unlike most other examples of symbiont-mediated immune priming, we find no evidence that *R. rhodnii* ever leaves the gut, despite activating a potent immune response in the hemocoel and fat body. *R. rhodnii* and *E. coli* activate both the IMD and Toll pathways indicating cross-activation of the pathways, while silencing of either pathway leads to a loss of the protective effect. Several antimicrobial peptides are induced in the fat body by presence of gut bacteria. When *E. coli* is in the gut, expression of antimicrobial peptides is often higher than when *R. rhodnii* is present, while *R. rhodnii* induces proliferation of hemocytes and induces a stronger melanization response than *E. coli*. Hemolymph from *R. rhodnii* bugs has a greater ability to convert the melanin precursor DOPA to melanization products than axenic or *E. coli*-harboring bugs. These results demonstrate that *R. rhodnii’s* benefits to its host extend beyond nutritional provisioning, playing an important role in the host immune system.

## Author summary

Insects often form beneficial relationships with bacteria allowing them to eat nutritionally deficient diets. In insects that only consume blood, symbionts are necessary to provide B vitamins absent in the host diet. There is a growing appreciation that in some of these symbiotic associations, the bacteria provide services beyond nutrition. We show that in kissing bugs, which feed exclusively on vertebrate blood and require bacterial symbionts for development, these symbiotic bacteria are important in activating the insect immune system. Insects with no gut bacteria are highly susceptible to infection and cannot mount an effective immune response. The bacteria reside exclusively in the insect gut yet protect against infections in the rest of the insect’s body. The bacteria must be alive to prime the immune system, and the response is dependent on the species of bacteria in the gut, with symbiotic bacteria providing stronger protection against infection and inducing a broader array of immune responses than commensal bacteria. This study expands our understanding of the role of beneficial bacteria in insect immunity and demonstrates that immune systems differ between major groups of insects.

## Introduction

Insect immunity is often presented as a set of discrete innate immune pathways targeting broad classes of pathogens. The Toll pathway acts against Gram-positive bacteria and fungi, the IMD pathway against Gram-negative bacteria, and the JAK/STAT pathway against viral infections [1]. These pathways are activated by microbe associated molecular pattern (MAMPs) and trigger humoral and cellular immune defenses to limit pathogen damage and proliferation. These insights have been gained primarily through studies undertaken in a small number of model systems, nearly all of which are holometabolous. Among the paurometabolous orders (insects that transition from a nymph to an adult without undergoing true metamorphosis), Hemiptera is the most diverse and arguably best studied, yet our understanding of the immune function in this group is far behind *Drosophila* and other holometabolous insects. Morphological, genomic, and experimental studies suggest that the immune systems of hemipterans are surprisingly different from holometabolous insects [2–4]. Morphologically, the hemipteran gut lacks the chitinous peritrophic matrix which forms a barrier between gut microbes and the midgut epithelium in most insect groups. In its place is the perimicrovillar membrane, a lipoprotein-based structure that has some similarities to the peritrophic matrix but is not entirely analogous [5]. The presence of the perimicrovillar membrane may promote different interactions between gut microbes and the host immune response relative to insects with a peritrophic matrix.

The first genome sequence of a hemipteran, the pea aphid *Acyrthosiphon pisum*, revealed that it lacks several genes in the IMD pathway thought to be essential for its function [6]. Subsequent hemipteran genomes have shown similar losses of core IMD genes, indicating these losses occurred early in the evolution of Hemiptera [4,7–9]. Functionally these two immune pathways are not as compartmentalized as in *Drosophila*, suggesting that these gene losses have significant consequences for the activation of the hemipteran immune system [4,10]. One explanation for the loss of these immune genes is that they are related to the frequent association of hemipterans with bacterial symbionts. Many hemipterans feed on nutritionally imbalanced diets and rely on microbial partners to supply the missing nutrients [11] and the need to support these symbionts may have led to a loss of immune genes that might activate host responses against these necessary microbes [12]. Despite the absence of canonical pathway components, many hemipterans can mount robust and effective immune responses [4,7,10,13–15].

While symbionts may have contributed to the loss of immune genes in hemipterans, they are increasingly appreciated for their role in host immune function in hemipterans and other insects. For example in the bean bug *Riptortus pedestris*, environmentally-acquired *Burkholderia* escape the midgut and subsequently prime the host immune response by stimulating AMP expression, resulting in increased survival following heterologous bacterial challenges [16,17]. This phenomenon is also seen in holometabolous insects. In honeybees (*Apis mellifera*), the presence of *Snodgrassella alvi* elevates production of the antimicrobial peptide (AMP) Apidaecin in the bee gut and provides protection against hemocoelic infection with *E. coli* [18]. In *Glossina* sp., when larvae are reared without their symbiont *Wigglesworthia glossinidia,* the resulting adults have impaired immune function, including reduced melanization, lower expression of AMPs, and reduced hemocyte titer [19]. Subsequent studies have demonstrated that *Wigglesworthia* are essential for hematopoiesis during *Glossina* development [20].

*Rhodnius prolixus*, a triatomine kissing bug (Hemiptera: Reduviidae), is a vector of *Trypanosoma cruzi*, a stercorarian parasite that causes Chagas disease in humans. *R. prolixus* harbors symbiotic Gram-positive Actinobacteria, *Rhodococcus rhodnii*, within the midgut which are essential for successful insect development [21–23]. A previous study demonstrated that gut bacteria can influence the host immune response to enteric pathogens, but surprisingly found that the immune system appeared to be minimally influenced by the presence of *R. rhodnii* [24]. That study employed antibiotic clearing of established communities of microbes from *R. prolixus* followed by recolonization with target microbes including *R. rhodnii*. This approach may have failed to clear non-target microbes from the gut, and systemic effects of antibiotics may have also influenced microbiota reestablishment and observed changes in immune function [25].

We previously developed a system for producing axenic (bacteria-free) nymphs without the use of antibiotics, which we leveraged to manipulate the host gut microbiome by re-introducing bacteria via a blood meal using artificial membrane feeders [22]. This experimental platform allows us to interrogate the effects of the symbiont on host physiology. We used this approach to ask if *R. rhodnii* had a significant impact on the *R. prolixus* immune system, whether other bacteria have similar effects to *R. rhodnii*, and what elements of the host immune system may be influenced by presence or absence of the symbiont. We show that *R. rhodnii* specifically is necessary for proper host immune function and involves both the Toll and IMD pathways. The symbiont activates both humoral and cellular immune responses in distal host tissues without leaving the insect gut, ultimately providing robust protection against hemocoel infection. These results expand our understanding of how symbionts contribute to host immune function and provide potential avenues to exploit towards limiting transmission of *T. cruzi*.

## Results

### The presence of bacteria protects *R. prolixus* against bacterial infection

**Fig 1:**
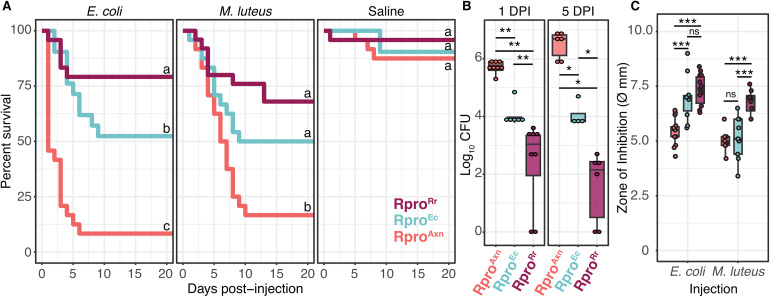
Gut bacteria promote immune priming against bacterial infection in *R. prolixus.* (A) Survival curves of Rpro^Axn^, Rpro^Ec^, and Rpro^Rr^ after injection with 10^6^ CFU of *E. coli, M. luteus,* or sterile saline into their hemocoel. Rpro^Rr^ and Rpro^Ec^ had significantly higher survival than Rpro^Axn^ regardless of the bacteria used for challenge (p < 0.0001, log-rank test), while there was no difference in survival of bugs injected with sterile saline (p ≥ 0.15, log-rank test) indicating that the presence of gut microbes plays a critical role in insect defense against pathogens. When challenged with *E. coli* there was a significant difference in survival (26.8%) between Rpro^Rr^ and Rpro^Ec^ (p = 0.018, log-rank test). The difference in survival between Rpro^Rr^ and Rpro^Ec^ was smaller (18%) and approached but did not reach significance when challenged with *M. luteus* (p = 0.072, log-rank test). Lines connected by different letters are significantly different (p < 0.05, log-rank test). (B) Gnotobiotic *R. prolixus* limit the growth of *E. coli* in the hemocoel. Boxplots *of E. coli* CFUs in *R. prolixus* hemolymph collected at 1 and 5 days post-infection (DPI). Points represent individual bugs. Rpro^Rr^ bugs had fewer *E. coli* CFU at both 1 and 5 DPI than Rpro^Ec^ or Rpro^Axn^. Rpro^Ec^ had fewer *E. coli* CFU at both 1 and 5 DPI than Rpro^Axn^ (** p < 0.002, * p < 0.05, Wilcoxon test). (C) Hemolymph from Rpro^Rr^ bugs suppresses growth of both *E. coli* and *M. luteus in vitro* more than Rpro^Ec^ or Rpro^Axn^ bugs. ***p < 0.001, Tukey’s HSD.

To investigate the effects of gut bacteria on host immune function, we removed all bacteria through surface sterilization of eggs using our previously described protocol [22]. Bugs were either reared in axenic conditions (Rpro^Axn^), or experimentally infected through a blood meal with *E. coli* MG1655 (Rpro^Ec^), or their symbiont *R. rhodnii* ATCC 35071 (Rpro^Rr^). Twenty four unfed 4^th^ instar nymphs from each gnotobiotic condition were injected with 10^6^ CFUs of *E. coli*, *R. rhodnii*, the Gram-positive *Micrococcus luteus,* or sterile saline into their thorax and survival after infection was monitored. Almost all Rpro^Axn^ individuals died within 5 days after injection with either *E. coli* (9% survival, Fig 1A) or *M. luteus* (17% survival, Fig 1A). In contrast, Rpro^Rr^ bugs showed significantly higher survival after *E. coli* or *M. luteus* infection (79% and 68% survival, respectively) than Rpro^Axn^ (p < 0.0001 for both pathogens, log-rank test). Rpro^Ec^ bugs had significantly higher survival rates compared to Rpro^Axn^ individuals for both challenges (52% survival, p < 0.0001, and 50% survival, p = 0.003, respectively, log-rank test, Fig 1A) and lower survival relative to Rpro^Rr^ challenged with *E. coli* (p = 0.018, log-rank test) but not when challenged with *M. luteus* (p = 0.07, log-rank test). To test if the mortality we saw was due to bacterial infection or due to wounding alone, we also injected Rpro^Axn^, Rpro^Ec^, and Rpro^Rr^ insects with sterile saline. All insects subject to sterile saline injection had high survival and were not significantly different from each other (p > 0.05, log-rank test, Fig 1A). We also tested whether lower infectious doses elicited the same pattern. We challenged Rpro^Rr^ and Rpro^Axn^ with 10^2^ and 10^4^ CFU of *E. coli* via the same injection protocol. We found no significant difference in survival between Rpro^Rr^ and Rpro^Axn^ at these lower doses which may reflect inoculations more frequently experienced by *R. prolixus* in nature (S1 Fig).

Our survival curves indicate that bugs reared with bacteria in their gut can survive infection with *E. coli* or *M. luteus* while nearly all Rpro^Axn^ die after infection. Insects can overcome infection through either resistance – killing or clearing of a pathogen – or through tolerance – minimizing fitness costs of infection without reducing the pathogen load. To determine if the observed difference in survival was due to tolerance or resistance, we measured *E. coli* titer in the hemolymph after infection over time. Because both the *E. coli* and *M. luteus* treatment groups had similar mortality rates we chose to further investigate only *E. coli* for this experiment. We injected 6–8 bugs with 10^6^ CFU of live, kanamycin-resistant *E. coli,* then collected hemolymph at 1- and 5- days post injection. Hemolymph was spread onto plates containing kanamycin to calculate CFUs of the kanamycin-resistant *E. coli* present in the hemolymph. The presence of bacteria in the gut had a significant impact on the number of Kan^R^ *E. coli* hemolymph (F_2,32_ = 123.2, p < 0.0001, aligned-rank transformed ANOVA). One day post injection with *E. coli*, Rpro^Rr^ insects had on average 1.6 x 10^3^ CFUs of *E. coli*, Rpro^Ec^ had 1.82 x 10^4^, and Rpro^Axn^ individuals had 4.6 x 10^5^ CFUs, higher than either gnotobiotic treatment (p = 0.002, Wilcoxon signed-rank test, Fig 1B). Five days after injection with *E. coli* most Rpro^Axn^ had died; those who remained alive had on average 4.3 x 10^6^ CFUs of *E. coli.* Rpro^Ec^ bugs had significantly fewer *E. coli* than Rpro^Axn^ bugs (1.8 x 10^4^ CFUs, p = 0.014, Wilcoxon signed-rank test, Fig 1B), while Rpro^Rr^ bugs had the least *E. coli* in their hemolymph of any treatment (2 x 10^2^ CFUs, p = 0.014, Wilcoxon signed-rank test). The identity of the gut bacteria influences the extent to which the gnotobiotic bugs can clear the bacteria from their hemolymph, with Rpro^Rr^ bugs having significantly fewer bacteria at day 5 than Rpro^Ec^ bugs (p = 0.014, Wilcoxon signed-rank test). Though neither gnotobiotic treatment completely cleared *E. coli* from their hemolymph during the observation window, they appear to suppress the pathogen to a level that is survivable.

We next sought to determine if the decrease in bacteria in the hemocoel of challenged Rpro^Axn^, Rpro^Ec^, and Rpro^Rr^ bugs was due to bacterial killing via antimicrobial factors in the hemolymph. To explore if presence of gut bacteria results in differences in antimicrobial activity in the bugs’ hemolymph, we conducted a zone of inhibition assay [26,27]. We injected nine Rpro^Axn^, Rpro^Ec^, and Rpro^Rr^ 4^th^ instar bugs with 10^6^ cells of either heat-killed *E. coli* or *M. luteus.* We used heat-killed bacteria for this assay to avoid bacterial growth on the plates that could potentially confound our zone of inhibition measurements. Rpro^Rr^ and Rpro^Ec^ bugs had significantly larger zones of inhibition when infected with *E. coli* than Rpro^Axn^ bugs (p < 0.0001, Tukey’s HSD, Fig 1C). Interestingly, only Rpro^Rr^ bugs had significantly larger zones of inhibition after being infected with *M. luteus* (p < 0.0001, Tukey’s HSD, Fig 1C). These results indicate that Rpro^Rr^ and Rpro^Ec^ bugs have higher antimicrobial activity in their hemolymph when challenged with Gram-negative *E. coli* while only Rpro^Rr^ bugs had higher antimicrobial activity after infection with the Gram-positive *M. luteus,* possibly due to activation of the Toll pathway in Rpro^Rr^. Naïve individuals did not show a significant difference in antimicrobial activity from each other, suggesting this is an induced response (S2 Fig). These data demonstrate the presence of gut bacteria promotes resistance to pathogens in *R. prolixus* through a reduction in the number of bacteria in the hemolymph of gnotobiotic bugs but not Rpro^Axn^ bugs.

The squash bug *Riptortus pedestris* harbors environmentally acquired *Burkholderia* bacteria that provide an immunoprotective effect to the host, which is induced upon migration from the M3 region of the midgut to the hemolymph [16,17]. We repeatedly isolated hemolymph from Rpro^Rr^ bugs and plated it on LB media that readily grows *R. rhodnii*. We screened dozens of Rpro^Rr^ yet did not observe any growth of *R. rhodnii* on plates, suggesting that *R. rhodnii* does not escape the gut, and that the immune priming effect of *R. rhodnii* occurs without direct contact with the insect hemocoel.

### The protective effect of *R. rhodnii* requires live bacteria and is lost upon removal

**Fig 2:**
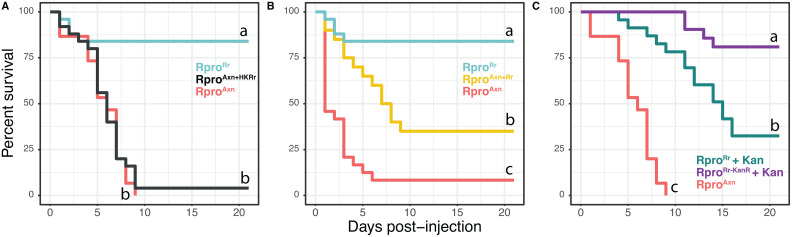
*R. prolixus* requires live bacteria to mount an effective immune response. (A) Survival after bacterial injection of 4^th^ instar Rpro^Axn^ nymphs fed heat-killed *R. rhodnii* throughout development (Rpro^Axn + HK Rr^) was similar to 4^th^ instar Rpro^Axn^ nymphs not fed bacteria (p = 0.69, log-rank test). Survival of Rpro^Rr^ was significantly higher (p < 0.0001, log-rank test). (B) Survival after immune challenge of Rpro^Axn^ bugs after restoration of *R. rhodnii* (Rpro^Axn + Rr^). Restoring *R. rhodnii* via a blood meal significantly increases survival relative to axenic nymphs (p < 0.0001, log-rank test) but there was still significantly lower survival in the Rpro^Axn + Rr^ than in Rpro^Rr^ (p < 0.0001, log-rank test). (C) Clearance of *R. rhodnii* from Rpro^Rr^ significantly reduced survival following immune challenge with *E. coli*. Rpro^Rr-KanR^ bugs treated with kanamycin retained their immune priming effect. Within a panel, lines not connected by the same letter are significantly different (p < 0.001, log-rank test). Each experiment consisted of 15–25 bugs per treatment.

Insects sense bacteria through detection of MAMPs such as peptidoglycan, lipopolysaccharides, and lipoteichoic acid, which activate signaling cascades via serine proteases. We therefore asked whether the immune priming effect of *R. rhodnii* was primarily due to the presence of MAMPs, and so we fed Rpro^Axn^ bugs blood meals that were spiked with an amount of heat-killed *R. rhodnii* that was equivalent to 10^8^ CFUs/bug (Rpro^Axn+HK Rr^) from the first blood meal until they developed into 4^th^ instars, then challenged them as before with injection of *E. coli*. All Rpro^Axn+HK Rr^ bugs died within the 10-day window and their survival was not statistically different from the survival of Rpro^Axn^ bugs (p = 1.00, Cox proportional hazards model, Fig 2A). From these results we conclude that live bacteria are likely necessary for immune priming in this system, though we cannot rule out a role for heat-labile small effector molecules produced by *R. rhodnii* in stimulating the immune response.

The main function of *R. rhodnii* is thought to be supplementation of B vitamins to *R. prolixus* [22,28]. These nutrients are important in numerous essential processes in the host and their absence throughout Rpro^Axn^ development may underlie the higher survival of Rpro^Rr^ following immune challenge. Alternatively, the protective effect of *R. rhodnii* may be independent of its nutrient provisioning services. If the effect were nutritional, we would expect that removal of *R. rhodnii* from Rpro^Rr^ bugs shortly before an immune challenge would not have a dramatic effect on survival, while if the effect were primarily immune priming, addition of *R. rhodnii* to Rpro^Axn^ bugs shortly before challenge would lead to increased survival relative to Rpro^Axn^ individuals.

To investigate this, we fed 3^rd^ instar Rpro^Axn^ nymphs a blood meal containing 10^6^ CFU/mL of *R. rhodnii*. Nymphs developed into 4^th^ instars and a subset were sacrificed and qPCR was used to confirm that sacrificed nymphs harbored *R. rhodnii* (S3 Fig). Two weeks after molting, insects were injected with live *E. coli* as previously outlined. Rpro^Axn+Rr^ bugs had higher survival than Rpro^Axn^ bugs (Fig 2B, p = 0.0047, Cox proportional hazards) but still suffered increased mortality compared to Rpro^Rr^ individuals. Interestingly, when we removed *R. rhodnii* from Rpro^Rr^ bugs by feeding kanamycin to 3^rd^ instar insects via the blood meal (Rpro^Rr ^+ Kan), they also suffered from increased mortality compared to bugs that were fed kanamycin but had been inoculated with kanamycin resistant *R. rhodnii* (Rpro^Rr-KanR ^+ Kan, Fig 2C). Thus, we conclude that the nutritional role of *R. rhodnii* in *R. prolixus* is not sufficient to explain the observed immune priming effects, but that nutrient provisioning may contribute to the protective effects of *R. rhodnii*. We did not test this in Rpro^Ec^ bugs, as their B vitamin synthesis capabilities are similar to *R. rhodnii*, and we do not anticipate that nutritional provisioning by *E. coli* would be able to rescue immune priming when *R. rhodnii* could not.

### Immune priming by gut bacteria acts through both the Toll and IMD pathways

**Fig 3:**
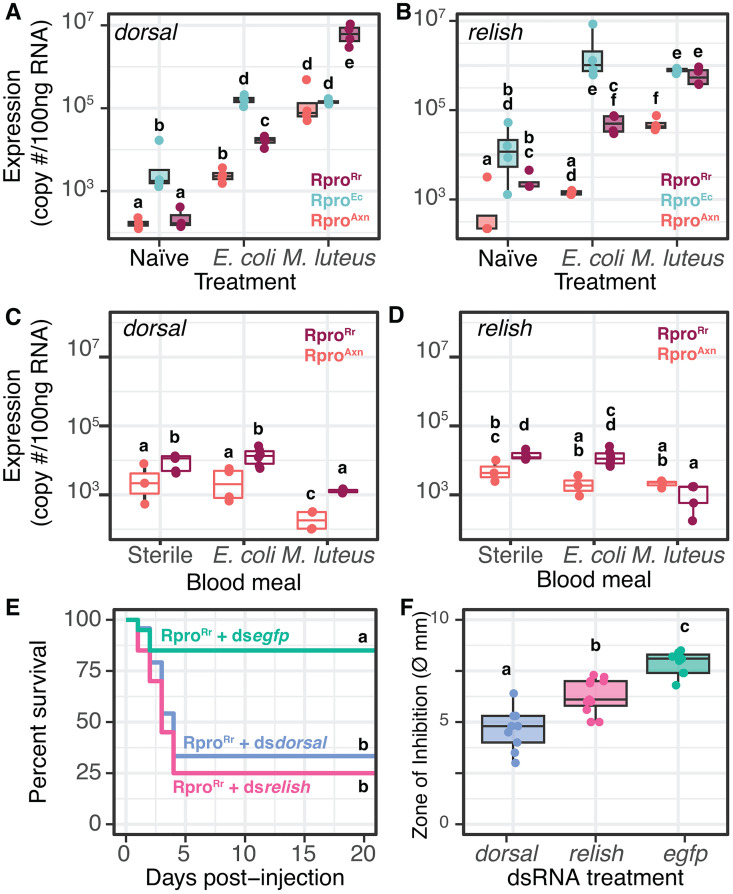
Immune priming by gut bacteria is dependent on Toll and IMD pathways. (A, C) Expression of the Toll pathway transcription factor *dorsal* in Rpro^Rr^, Rpro^Ec^, and Rpro^Axn^ in naïve bugs or bugs challenged with *E. coli* or *M. luteus* (A) injection or (C) via an inoculated blood meal. (B, D) Expression of the IMD pathway transcription factor *relish* in Rpro^Rr^, Rpro^Ec^, and Rpro^Axn^ in (B) naïve bugs or bugs challenged with *E. coli* or *M. luteus* injection or (D) orally via an inoculated blood meal. Bars connected by different letters are significantly different (p < 0.05, Tukey’s HSD). (E) Survival curves of Rpro^Rr^ bugs treated with dsRNA against *dorsal, relish*, or a control *egfp* sequence demonstrate that silencing of *relish* or *dorsal* via RNAi significantly reduced the survival of Rpro^Rr^ bugs, indicating that these pathways are necessary for *R. rhodnii*-mediated immune priming. Different letters indicate treatments with significantly different survival (p < 0.0001, log-rank test). (F) Silencing *dorsal* or *relish* reduces the bacteriostatic factors in hemolymph of Rpro^Rr^. Different letters indicate treatments with significantly different survival (p < 0.0001, Tukey’s HSD).

Insect immune responses to bacteria are thought to be mediated by two pathways, Toll and IMD [29]. Our zone of inhibition assays suggested that some factor(s) in the hemolymph are involved in suppressing bacterial proliferation, which may be regulated by Toll or IMD. To investigate whether the Toll or IMD pathways are essential to *R. rhodnii*-mediated immune priming, we first measured expression of two transcription factors, *dorsal* and *relish*, which drive expression of Toll and IMD response genes including antimicrobial peptides (AMPs). For both genes, we assessed expression via qPCR in insects injected or blood-fed with live *E. coli* or *M. luteus*, as described above. We tested the fat bodies of naïve or injected insects and the guts of blood-fed insects. We tested Rpro^Rr^, Rpro^Ec^, and Rpro^Axn^ bugs, and found that naïve, Rpro^Axn^ bugs had low expression of both *dorsal* and *relish* (Fig 3A and 3B).

For each gene, there was a highly significant effect of the gnotobiotic state of the bugs, the immune challenge, and the interaction between state and challenge (see S1 Table for statistical details). Naïve Rpro^Ec^ bugs had higher expression of *dorsal* and *relish* than Rpro^Axn^, indicating that *E. coli* in the gut stimulates expression of both transcription factors independent of immune challenge via injection. Naïve Rpro^Rr^ bugs had low expression of *dorsal*, equivalent to Rpro^Axn^ bugs but had higher expression of *relish* than Rpro^Axn^. In bugs challenged with injection of *E. coli*, both *dorsal* and *relish* expression were elevated in their respective gnotobiotic bugs relative to Rpro^Axn^. *M. luteus* also stimulated robust expression of *relish* and *dorsal*, with Rpro^Rr^ bugs having the strongest expression of *dorsal* while *relish* expression was high in both Rpro^Rr^ and Rpro^Ec^. Feeding of Rpro^Rr^ and Rpro^Axn^ with *E. coli* or *M. luteus* induced a smaller change in expression of *relish* or *dorsal*, though for most treatments, Rpro^Rr^ had significantly higher gut expression of these genes than Rpro^Axn^ (p < 0.05, Tukeys HSD, Fig 3C and 3D).

From our expression analysis of *dorsal* and *relish*, we observe significant cross activation of the Toll and IMD pathways, with both Gram-negative and Gram-positive bacteria activating both pathways. The Toll pathway, and by extension *dorsal*, is thought to primarily respond to Gram-positive bacteria while IMD and *relish* are thought to be activated by Gram-negative bacteria, but significant crosstalk between these pathways has been observed in other hemipterans [4]. Absence of gut bacteria does not eliminate the ability of bugs to mount an immune response, but gnotobiotic bugs almost always have higher expression of these genes. In all bugs tested, both *dorsal* and *relish* can be induced by the presence of bacteria in the hemolymph, but we were surprised to see that Rpro^Ec^ often induces higher expression of *dorsal* and *relish* than Rpro^Rr^, given the latter’s stronger protective effect.

We next asked whether the immune priming effect of gut bacteria is dependent on the Toll or IMD pathway. We suppressed each pathway via RNAi knockdown of the transcription factors *relish* (IMD) or *dorsal* (Toll). RNAi of *relish* or *dorsal* was confirmed via qPCR revealing over a 90% reduction in *dorsal* or *relish* transcripts after injection with dsRNA (S4 Fig). After injection with dsRNA, 10^6^ CFUs of *E. coli* were injected into the bugs’ hemocoel, and survival rates were measured (Fig 3C). Rpro^Rr^ treated with either *dorsal* or *relish* dsRNA succumbed to *E. coli* infection at a higher rate than Rpro^Rr^ individuals treated with ds*egfp* (25% and 34% survival respectively, p = 0.002, p = 0.0004, respectively, log-rank test).

We then examined the change in antimicrobial activity in *relish* or *dorsal*-silenced *R. prolixus* by comparing the inhibitory effects of hemolymph on microbial growth using the zone of inhibition assay described above, though we only tested the effect of hemolymph from dsRNA-treated bugs against *E. coli*. There was a significant effect of silencing either *relish* or *dorsal* as silenced bugs had smaller zones of inhibitions compared to the control ds*egfp*-injected group (Fig 3D, p < 0.0007, p < 0.0001, Tukey’s HSD). The decrease in survival after challenge with *E. coli* in either *relish* or *dorsal* knockdown bugs suggests immune pathway cross activation in *R. prolixus*, as silencing of the Toll pathway transcription factor *dorsal* led to significantly higher mortality following challenge with Gram-negative bacteria. The reduction in antimicrobial activity in hemolymph following knockdown of *dorsal* or *relish* suggests that the activation of the Toll and IMD pathways by *R. rhodnii* has functional consequences for host immune responses.

### *R. rhodnii* influences the cellular immune system

**Fig 4:**
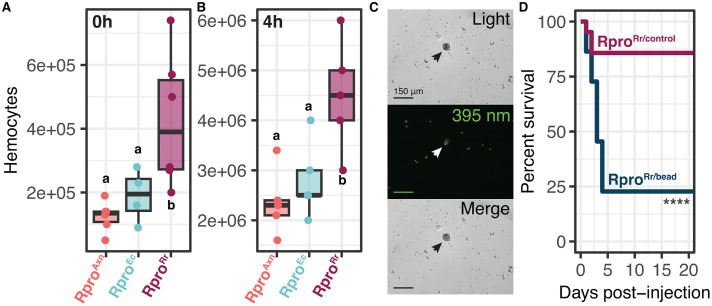
Rpro^Rr^ bugs have higher baseline and induced numbers of hemocytes relative to Rpro^Ec^ and Rpro^Axn^ bugs. (A) Hemocyte counts from hemolymph extracted at 0 h post-challenge with *E. coli*. There was no significant difference in the number of hemocytes between Rpro^Axn^ and Rpro^Ec^ but Rpro^Rr^ had significantly more than either (p = 0.0001, p = 0.02, Tukey’s HSD). (B) At 4 h post challenge, all bugs had more hemocytes but Rpro^Rr^ still had significantly more than Rpro^Ec^ or Rpro^Axn^, which were not significantly different from one another (p = 0.0008, p = 0.001, p = 0.23 respectively, Tukey’s HSD). (C) Fluorescent latex beads are consumed by a hemocyte (arrow) in Rpro^Rr^. Top panel: light microscopy, middle panel: 395 nm, bottom panel: merge. (D) Hemocytes are essential for *R. rhodnii*-mediated immune effects. Inactivation of hemocytes by injection of latex beads dramatically reduced survival of Rpro^Rr^ bugs after challenge with *E. coli* (**** p < 0.0001, log-rank test).

Cellular immune responses are often the first line of defense against pathogens, clearing many bacteria from the hemolymph before upregulation of antimicrobial peptide gene expression [26]. Given the robust immunoprotective effect of *R. rhodnii*, we wondered if presence of *R. rhodnii* induced cellular immune responses. We first measured the number of circulating hemocytes in the hemolymph of five Rpro^Rr^, Rpro^Ec^, and Rpro^Axn^ bugs. Fourth instar nymphs were immune challenged with *E. coli* and hemolymph was collected at 0 and 4 hours post-injection and a hemocytometer was used to count the number of hemocytes present. Immediately after injection Rpro^Rr^ bugs had a significantly higher number of circulating hemocytes compared to Rpro^Axn^ bugs (p = 0.0001, Fig 4A) and Rpro^Ec^ bugs (p = 0.02, Tukey’s HSD) and there was no significant difference in hemocyte counts between Rpro^Axn^ and Rpro^Ec^ (p = 0.11, Tukey’s HSD). These results demonstrate that Rpro^Rr^ bugs have a higher initial number of hemocytes than Rpro^Ec^ or Rpro^Axn^. Four hours after injection, all hemocyte titers had increased (Fig 4B, p < 0.001 for all comparisons, Tukey’s HSD), but Rpro^Rr^ insects still had significantly higher hemocyte counts than both Rpro^Axn^ and Rpro^Ec^ bugs (p = 0.0008 and p = 0.001, respectively, Tukey’s HSD).

To investigate whether the differences in hemocyte counts among the treatments were a major factor in infection outcome, we suppressed hemocyte phagocytosis by pre-injecting fluorescent latex beads into the hemocoel of Rpro^Rr^ bugs [30]. The beads were visually confirmed to be phagocytosed by the hemocytes *in vitro* via microscopy (Fig 4C). Twenty-four hours after bead injection, *E. coli* was injected into the hemolymph and survival was monitored as previously described. Insects with suppressed cellular immunity (Rpro^Rr/bead^) succumbed to bacterial infection faster than Rpro^Rr/control^ insects (p < 0.05, log-rank test, Fig 4D), indicating that *R. rhodnii*-mediated cellular immunity represents an important component of *R. rhodnii*-based immune priming, but that *E. coli* in the gut does not induce as strong of a cellular response.

### Expression of immune-related genes is influenced by the gut microbiome

**Fig 5:**
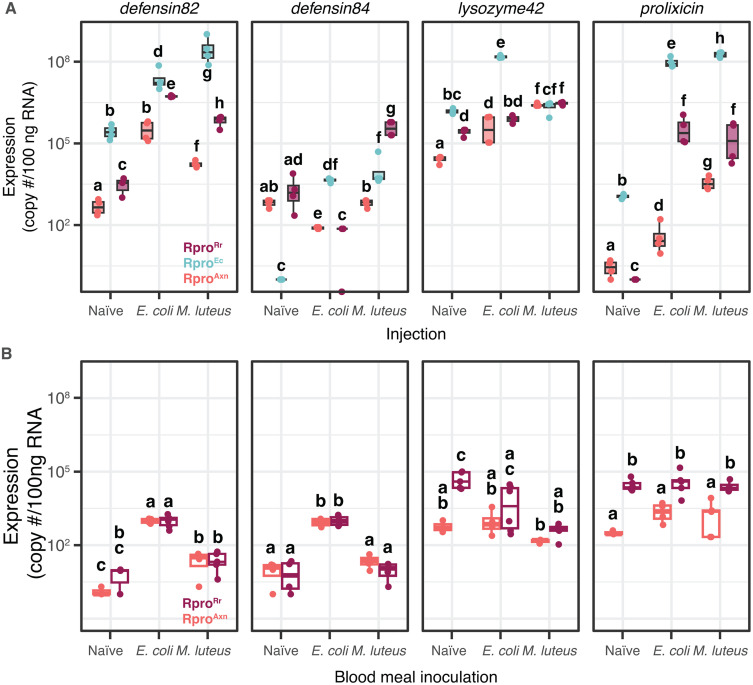
Antimicrobial peptide (AMP) genes are differentially expressed among naïve, Rpro^Ec^, and Rpro^Rr^ bugs. Induction of AMP expression was generally higher in insects (A) injected with bacteria than in insects that were (B) fed blood meals inoculated with bacteria. Within each AMP panel, bars connected by different letters are significantly different (p < 0.05, Tukey’s HSD).

Our experiments demonstrated that *dorsal* and *relish* were important mediators of the immune priming effect seen in Rpro^Rr^. We next wanted to see if antimicrobial peptides were upregulated in gnotobiotic insects. To test this, we evaluated the fat body expression profiles of AMPs in 4^th^ instar Rpro^Axn^, Rpro^Ec^, and Rpro^Rr^ bugs that were either uninfected or 1-day post-inoculation with either *E. coli* or *M. luteus*. We measured the expression of the AMPs *prolixicin*, two defensins (RPRC012182 and RPRC012184, subsequently referred to as *defensin82*, and *defensin84*), and a lysozyme (RPRC015442 subsequently referred to as *lysozyme42*, Fig 5A).

The interaction of gnotobiotic state by injection was highly significant for all genes tested (p < 0.0001, aligned ranks transformation ANOVA for non-parametric interactions). Our expression analysis of immune genes revealed several interesting patterns. First, Rpro^Axn^ insects often had lower expression of AMP genes regardless of the immune challenge (Fig 5A), which may be a consequence of lower *relish* and *dorsal* expression in Rpro^Axn^ bugs (Fig 3A and 3B). The overall reduced expression of immune genes may partially explain the heightened susceptibility of Rpro^Axn^ to pathogens. A second, surprising pattern is that Rpro^Ec^ bugs often have higher expression of AMPs than Rpro^Rr^ bugs, despite Rpro^Rr^ having a significantly higher survival rate when challenged with pathogens. In the Rpro^Ec^ bugs challenged with *E. coli*, expression of all immune genes except *defensin84* were significantly higher than when Rpro^Rr^ bugs were challenged with *E. coli*, indicative of a strong immune priming effect of *E. coli* in the gut against *E. coli* in the hemolymph (Fig 5A, p < 0.05, Tukey’s HSD). Rpro^Rr^ bugs exhibited strong induction of immune gene expression in response to bacterial challenge relative to naïve Rpro^Rr^ or Rpro^Axn^, apart from *defensin84*, suggesting that humoral immune responses are important in the heterologous immune priming seen in Rpro^Rr^ following pathogen challenge. Oral challenge with *E. coli* induced expression of AMPs *defensin82* and *defensin84*, but the induction was independent of gnotobiotic state. Rpro^Rr^ had higher expression of *prolixicin* than Rpro^Axn^ bugs, but this was independent of pathogen challenge (Fig 5B).

The third pattern we observed was further evidence of cross activation of the Toll and IMD immune pathways, consistent with the earlier experiments examining *dorsal* and *relish* (Fig 3). Defensins, AMPs which act primarily against Gram-positive bacteria [31], were highly expressed in Rpro^Ec^ in response to challenge with Gram-negative bacteria (Fig 5, *defensin 84, 82*). Likewise, *lysozyme42* expression was also induced by Gram-negative bacteria (Fig 5A) despite canonically being considered an AMP against Gram-positive bacteria [32]. A similar pattern was seen with expression of *prolixicin*, an ortholog of *Drosophila diptericin,* as it was induced in Rpro^Rr^ bugs after challenge with *M. luteus*. This is despite Diptericin being active against Gram-negative bacteria [33]. Our data supports and expands on earlier work in the stink bug *Plautia stali* which demonstrated that expression of *relish* and several immune effectors can be induced by both *E. coli* and *M. luteus* in Hemiptera [4].

### Melanization and phenol oxidase activity are dependent on the presence of *R. rhodnii*

**Fig 6:**
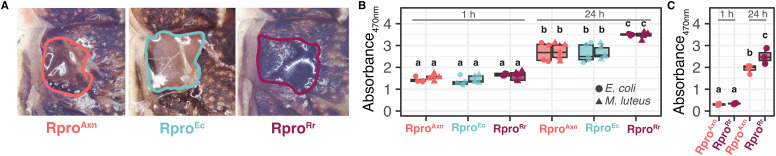
*R. rhodnii* enables successful melanization in *R. prolixus.* (A) Melanization of wounds is impaired in Rpro^Axn^ and Rpro^Ec^ relative to Rpro^Rr^. Wound area is outlined. (B) Hemolymph DOPA conversion assay of Rpro^Axn^, Rpro^Ec^, and Rpro^Rr^ measured at 1 and 24 h post-hemolymph collection with E. coli (circles) or M. luteus (triangles). At 1 h, there was no significant difference in conversion of DOPA to melanin between the bugs or bacterial challenges (F_2,44_ = 0.11 p = 0.74, ANOVA). By 24 h post-collection, there was a significant increase in DOPA conversion in all treatments relative to 0 h (p < 0.05, Tukey’s HSD). Rpro^Rr^ bugs had higher DOPA conversion than Rpro^Axn^ and Rpro^Ec^ bugs (p < 0.05, Tukey’s HSD), which were not significantly different from each other (p > 0.05, Tukey’s HSD). As with the 1 h time point, there was no significant effect of the bacterial species used in the challenge (F_1,44_ = 0.21, p = 0.648, ANOVA). (C) Wounding induces activation of the melanization response 24h post-hemolymph collection. Rpro^Rr^ had higher DOPA conversion in response to wounding than Rpro^Axn^. Bars connected by different letters are significantly different (p < 0.05, Tukey’s HSD n = 5 insects per treatment).

We observed throughout our experiments that Rpro^Axn^ individuals exhibited reduced wound healing. Rpro^Axn^ bugs did not develop a robust, dark melanization scar at injection sites but rather a light, thin scar, while Rpro^Rr^ developed a characteristic thick, dark, scar (Fig 6A). Interestingly we also saw a lack of dark, thick scar tissue in Rpro^Ec^ bugs. The dark scars following wounding are due to deposition of melanin produced by the action of phenol oxidases and other enzymes in the hemolymph which convert tyrosine to melanin [34]. The cascade leading to melanization can be triggered by both wounding and MAMPs [35]. Based on these observations and the role of melanization in insect immunity, we decided to further investigate differences in melanization in Rpro^Rr^, Rpro^Ec^, and Rpro^Axn^ insects.

We measured phenol oxidase activity via a dihydroxyphenylalanine (DOPA) conversion assay [36] to assess how the presence of gut microbes influences melanization. DOPA is a precursor metabolite that is converted into melanin via the action of phenol oxidases. Hemolymph DOPA conversion was measured at 1 and 24 hours following hemolymph collection. There was a highly significant effect of the gnotobiotic state (Fig 6B, F_2,54 _= 23.8, p = 3.88 x 10^-8^, ANOVA) and time (F_1,54 _= 681, p < 2 x 10^-16^, ANOVA) on the amount of DOPA conversion. Surprisingly, there was not an effect of the microbe injected, as both *E. coli* and *M. luteus* injection triggered similar melanization among the different gnotobiotic states (Fig 6B, F_3,54_ = 0.09, p = 0.96). There was no significant difference in DOPA conversion at 1 h post-collection, but at 24 h post-collection there was a significant increase after injection of either *E. coli* or *M. luteus*. Compared to Rpro^Ec^ and Rpro^Axn^, Rpro^Rr^ had greater DOPA conversion regardless of challenge. We also investigated whether a stab wound alone was sufficient to trigger an increase in melanization. We stabbed Rpro^Axn^ and Rpro^Rr^ bugs with a sterile insulin syringe, then collected hemolymph and measured DOPA conversion as before at 1 and 24 h post-collection. We found that similar to injections with *E. coli* or *M. luteus*, stabbing with a sterile needle induced the ability to convert DOPA in both Rpro^Axn^ and Rpro^Rr^ hemolymph by 24 h (Fig 6C, p < 0.001, Tukey’s HSD) but not 1 h (p = 0.99, Tukey’s HSD), with a larger increase in conversion seen in the Rpro^Rr^ bugs (p = 0.014, Tukey’s HSD). Our DOPA conversion assays suggest that *R. rhodnii* is important for successful melanization in *R. prolixus*.

## Discussion

We demonstrate that the presence of gut microbes is integral to a functional immune system in *R. prolixus* and key to their ability to overcome infection with facultatively pathogenic microbes. We provide multiple lines of evidence that gut microbes in *R. prolixus* induce a strong immune response against hemocoelic pathogens, “priming” the immune system by influencing humoral and cellular immunity, and that symbiotic *R. rhodnii* induce a stronger priming and protective effect than commensal *E. coli*. This enhanced immune response encompasses both cellular and humoral immunity and is active against diverse bacterial pathogens, suggesting a hyperactivation of immune responses by the symbiont *R. rhodnii*. Bugs that harbored a microbe in their gut exhibited a strong humoral immune response by expressing significantly higher levels of antimicrobial peptides than microbe-free Rpro^Axn^ individuals, that corresponded to higher antimicrobial activity in their hemolymph, and subsequently an increased ability to reduce the number of infecting microbes compared to Rpro^Axn^ bugs. Our results suggest that different gut microbes activate the immune system in different ways and to different extents. *R. rhodnii* produces a stronger effect and modulates both cellular and humoral immunity while *E. coli* mainly stimulates humoral immune factors and does not have as strong of a priming effect.

Immune priming has been revealed to be a broadly important facet in insect immunity. Despite their lack of antibody-mediated adaptive immunity, many insect species exhibit elevated immune responses after a second exposure to a nonlethal dose of a pathogen or pathogen derived material. This priming manifests as increased production of AMPs and mobilization or proliferation of circulating hemocytes [37], ultimately leading to the insect being resistant to subsequent infections [16,38–41]. Immune priming was initially characterized through experiments where an insect is repeatedly exposed to a single pathogen, known as homologous immune priming [42]. More recently, heterologous immune priming, in which one pathogen induces a protective immune response towards a different secondary pathogen has been observed [38]. The growing appreciation for symbiotic associations between insects and microbes has also illuminated the role of symbionts in host immune function [13,16–20,43,44]. Our results suggest that both the Gram-positive symbiotic *R. rhodnii* and Gram-negative commensal *E. coli* stimulate heterologous immune priming against a different bacterial species, though to varying degrees.

In other systems where symbiont-mediated immune priming has been observed, the bacteria responsible have at least some contact with the host hemolymph or extracellular tissues beyond the gut. Tsetse flies (*Glossina* sp.), which are also obligately exclusively hematophagous, harbor an intracellular symbiont, *Wigglesworthia glossinidia*. The symbiont is essential for proper immune function in *Glossina morsitans*, as aposymbiotic larvae develop into adults with impaired immune responses, reduced hemocyte proliferation, and inability to melanize properly [19,20,43]. To achieve an immune priming effect, *Wigglesworthia* must be present during larval development [19,20,43]. During this time *Wigglesworthia* can be found in the lumen of the mother’s milk glands and extracellularly in larval fat body [45]. In the bean bug, *Riptortus pedestris,* their gut symbiont *Caballeronia,* which resides in specialized crypts within the gut, strongly contributes to the activation of host immunity [17]. However, stronger immune priming is seen when a soil-derived *Burkholderia* colonizes *R. pedestris* and escapes the gut, increasing AMP expression and hemocyte number [16]. The priming observed in *Riptortus* by *Burkholderia* sp. involves extracellular localization of the bacterium allowing for direct contact with the host hemolymph and presumably hemocytes [16].

Mosquito immunity has also been shown to be influenced by symbiotic bacteria. *Wolbachia* upregulates immune-related genes of its natural *Drosophila* host as well as when artificially introduced to mosquitoes, thereby conferring protection against a variety of pathogens [46–50]. Though *Wolbachia* is nearly always intracellular, it can infect a variety of insect tissues including the immunologically important fat body [46]. The mosquito symbiont *Asaia* activates AMP production in *Anopheles stephensi* mosquitos, and though primarily associated with the gut, it is found in several other tissues [51].

In contrast to the *Glossina-Wigglesworthia, Riptortis-Burkholderia*, and *Aedes-Wolbachia* or *Asaia* systems, we never detected *R. rhodnii* outside of the gut of *R. prolixus*. *R. rhodnii* is environmentally acquired each generation and does not escape the insect’s alimentary canal. Despite this, it potentiates a strong and multifaceted immune response beyond the insect gut. We observe mobilization of hemocytes, increased expression of AMPs in the fat body, and elevated melanization potential in the hemolymph. We initially hypothesized that *R. rhodnii* MAMPs may escape the gut and activate the host immune system, and thus expected dead *R. rhodnii* would be able to recapitulate Rpro^Rr^ immune priming. In other systems, dead bacteria can elicit immune priming [30], but dead bacteria did not stimulate a protective response. We are left to conclude that *R. rhodnii* produces some extracellular signal or activates a host signaling mechanism in the gut that reaches the fat body and hemocytes to induce immune protection. Several candidate molecules including nitric oxide and prostaglandins have been previously shown to be important in *R. prolixus* immunity [24,52,53], and may be necessary for symbiont mediated immune priming.

Differences in the gut anatomy and physiology of kissing bugs may contribute to the ability of *R. rhodnii* to activate the host immune system without leaving the gut. Most insects possess an acellular chitinous and proteinaceous peritrophic matrix that lines the midgut epithelium and is responsible for protecting the midgut cells from direct contact with gut microbes. This protective barrier modulates immune activation by the gut microbiome [54]. Hemipterans, including kissing bugs, do not have a peritrophic matrix (PM), but rather a lipid-based structure called the perimicrovillar membrane (PMM). While the PMM forms a barrier between the gut lumen and its resident microbiota, it is possible that the PMM is not as significant a barrier to microbes or MAMPs and may permit direct contact of gut microbes with the gut epithelia. Such direct contact could potentially activate host immune responses to a greater extent than if bacteria did not contact the epithelial cells directly [5]. Additional studies are necessary to understand the extent to which gut bacteria or MAMPs in *R. prolixus* encounter the epithelium, and how this influences immune responses.

The immune priming response we observed in Rpro^Rr^ was very strong in comparison to similar experiments in *Drosophila,* which used lower doses of bacteria for immune challenges. Immune priming by prior exposure to *Enterococcus faecalis* lead to ~30% of challenged flies surviving a low dose infection with ~3,000 CFU of *E. faecalis* and only ~25% of flies surviving a high dose infection of 30,000 CFU [55]. *Drosophila* primed against *Streptococcus pneumoniae* had higher survival against 250 or 35,000 CFU of live *S. pneumoniae* [30]. Another study using heat-killed *Providencia rettgeri* followed by challenge with > 100 CFU of live bacteria found variable protection from subsequent infections even at this low dose [56]. In our experiments Rpro^Rr^ exhibited high survival rates (65% to 75%) when challenged with 10^6^ CFU of *E. coli* or *M. luteus,* demonstrating that the immune priming effect of *R. rhodnii* in *R. prolixus* is highly effective at protecting the host from these infections. The ability to survive challenge with higher bacterial loads may be related to the specific pathogens tested. The priming observed in the *E. faecalis* experiments was attributed primarily to tolerance, as primed flies had higher bacterial loads at death than un-primed flies [55]. In the experiments using *P. rettgeri*, both primed and un-primed insects displayed clearance of the pathogen, suggesting that the clearance was independent of priming [56]. Again, our results highlight differences between *R. prolixus* and *Drosophila*, as Rpro^Rr^ were able to suppress and reduce the number of bacteria following challenge, indicating that Rpro^Rr^ survival is related to resistance to pathogens rather than tolerance.

Though many bacterial pathogens likely invade through ingestion and subsequently escape the midgut, we chose to focus our experiments on injection directly into the hemocoel. Recent work has demonstrated that up to 30% of wild-caught insects have scars that cannot be explained by parasitism by wasps or mites, demonstrating that injury is common in insects [57]. Much like vertebrates, the risk of infection following injury is likely significant, as insects have evolved a robust and rapid immune response to wounding that involves activation of insect immune pathways, mobilization of hemocytes, and production of AMPs [58–61]. Injuries can lead to resulting infections, as seen in parasitic mite bites which lead to secondary bacterial infections in *A. mellifera* [62], and in *Drosophila* where hemolymph-feeding mites transfer pathogens between flies [63]. Kissing bugs may be particularly susceptible to injury and subsequent infection as they exhibit kleptohematophagy, where bugs will attack conspecifics and steal blood from fed individuals [64]. Furthermore, they are long-lived relative to many other model insects, potentially increasing the likelihood of injury during their lifetime [65,66]. A second rationale for introduction of pathogens via injection over feeding is to isolate the effects of the immune system from confounding factors that would have influenced a feeding-based infection. These factors include direct and indirect bacterial competition between gut bacteria and those being used for immune challenge, niche exclusion of bacteria used for challenges, and vertebrate immune proteins in the blood meal.

While wounding is likely an underappreciated route for infection in insects, it is unlikely that insects such as *R. prolixus* would experience an inoculation of the same magnitude as we employed in our experiments. We chose a titer of bacteria that induced significant mortality in axenic but not gnotobiotic insects which clarified the fitness effects of gut symbionts in relation to infection. While we did not observe significant mortality in experiments with lower inoculations of bacteria, we did not follow up with subsequent studies of the outcomes of these infections regarding long-term survival, successful development, or reproduction. More field-relevant inoculations may still have significant but subtler fitness effects that our experiments were not designed to capture. However, the protective effects of *R. rhodnii* against high titers of pathogens suggests that the symbiont likely would reduce the fitness costs of field-realistic infections.

Studies of immune priming in insects examined several insect lineages including *Drosophila melanogaster* [67,68], *Aedes aegypti* [69], *Anopheles sp.* [70–72], *Tenbrio* [73], *Gallaria mellonella* [74], *Tribolium castaneum* [75] among others. These studies have primarily explored holometabolous insects and relatively few have been performed in paurometabolous orders. Our results contribute to a growing understanding that hemipteran immunity is substantially different from many holometabolous model organisms such as *Drosophila*. Genomic data has revealed that many hemipteran genomes lack elements of the canonical insect immune pathways, including aphids [2], bedbugs [76], scale insects [77], and plant hoppers [78]. The genome sequence of *R. prolixus* was initially thought to be missing key components of the IMD pathway, though subsequent analysis has revealed that despite lack of the genes *imd* and *kenny*, *R. prolixus* does indeed possess a functioning IMD pathway [3,79]. Our results support these earlier findings, as both *R. rhodnii* and *E. coli* stimulate expression of *relish* and IMD-associated AMPs, while inactivation of IMD signaling through RNAi silencing of *relish* leads to increased mortality of *R. prolixus.* The absence of *imd* and *kenny* suggests that IMD immune signaling functions differently in *R. prolixus* highlighting the need for further investigation into immune signaling in *R. prolixus* and hemipterans in general.

Immune signaling in insects was initially described in *Drosophila* as a linear response where different classes of pathogens trigger discrete immune pathways: Gram-negative bacteria activate the IMD pathway, while Gram-positive bacteria and fungi activate the Toll pathway [29]. Yet as insect immune studies have moved beyond *Drosophila* and into other insects, it appears that crosstalk between or cross activation of immune pathways may be more common than previously thought. Work in the hemipteran stink bug, *Plautia stali*, revealed that not only are both IMD and Toll pathways present but there is a blurred functional differentiation, as immune challenge with Gram-negative or Gram-positive bacteria elicited expression of immune effector genes of both pathways [4]. We see similar patterns in our expression data as immune challenge with Gram-positive and Gram-negative bacteria trigger expression of both *dorsal* and *relish* immune transcription factors belonging to the Toll and IMD pathway respectively. Knockdown of either *dorsal* or *relish* also results in increased mortality after challenge with Gram-negative *E. coli,* and AMPs thought to be active against either Gram-positive or Gram-negative bacteria are expressed in response to both Gram-negative and positive bacteria. Crosstalk has been seen in other insects, including the beetle *Tenebrio molitor* [73], and other species of kissing bugs. In *Triatoma pallidipennis,* silencing of Toll pathway genes led to increased mortality when bugs were challenged with Gram-negative bacteria [10]. Interestingly, silencing of *relish* in *T. pallidipennis* did not result in increased mortality. Even in *Drosophila*, gut bacteria can elicit immune priming against heterologous pathogens including fungi [30] and viruses [80]. Together, these studies and others broaden our understanding of insect immunity, regarding both the Toll and IMD signaling pathways and immune priming by heterologous bacteria.

While *R. rhodnii* appears to be a ubiquitous member of the gut community in members of the genus *Rhodnius* [23], surveys of the microbiome of wild-caught *Rhodnius pallescens* describe a moderate diversity community that varies with age, geographic location, and *T. cruzi* status [81]. Our experiments were performed in a long-term lab colony of *R. prolixus* maintained at the Centers for Disease Control and Prevention since at least 1989 (Ellen Dotson, personal communication). At both the CDC and in our lab, the colony is maintained on sterile defibrinated rabbit blood supplemented with *R. rhodnii* using artificial membrane feeders [82]. This has likely altered the microbiome of the bugs, as other lab-reared triatomines have distinct microbiomes relative to wild-caught individuals [83]. While our previous results demonstrate that *R. rhodnii* in isolation is a necessary and sufficient symbiont of *R. prolixus* [22] and the current study demonstrates that *R. rhodnii* provides robust immune priming to *R. prolixus*, it is possible that other microbes in the *R. prolixus* gut influence the insect’s immune response or alter the impact of *R. rhodnii* on host immune function. Our findings that Rpro^Ec^ individuals had higher expression of several AMP genes tested when compared to Rpro^Rr^ bugs demonstrate that different gut microbes have different modes of immune priming of *R. prolixus*. The natural, more complex microbiomes of wild triatomines may induce even stronger protection against pathogens than *R. rhodnii* alone does for *R. prolixus*.

Immune function has been linked to nutrition in many different insects. Starving or rearing insects on nutritionally poor diets alters the humoral and cellular immunity leading to increased mortality after infection [84]. Diet interacts with the immune response in *R. prolixus* as well. Thirty days of starvation post-ecdysis resulted in increased mortality after infection due to changes in the cellular immune system, and similar results were found when bugs were fed an incomplete diet of plasma alone [85]. All bugs used in our experiments were two weeks post-molt to control for any effects starvation has on survival after infection. We further attempted to disentangle the impacts of nutrition on survival by clearing *R. rhodnii* from Rpro^Rr^ with a blood meal containing antibiotics. These cleared bugs were confirmed to have no *R. rhodnii* present and were challenged with *E. coli*. Interestingly, these bugs suffered high mortality rates, though not as high as Rpro^Axn^ bugs, suggesting that *R. rhodnii*’*s* influence on the immune system is not primarily mediated by nutritional factors. Conversely, axenic individuals that were fed a blood meal containing *R. rhodnii* at their 3^rd^ instar then challenged with *E. coli* shortly after had higher survival than Rpro^Axn^ but significantly lower survival than our Rpro^Rr^ group. These results taken together suggest that nutritional supplementation via the microbiome may play some role in immunity, but other factors are likely more important than this interaction. Direct experiments with B vitamin supplementation and B vitamin auxotrophic *R. rhodnii* will be necessary to fully resolve the role of symbiont-provisioned nutrients in *R. prolixus* immunity.

Kissing bugs are the vectors of *Trypanosoma cruzi*, the causative agent of Chagas disease. *T. cruzi* is a stercorarian parasite, residing exclusively in the gut of its triatomine host during the insect phase of its development. As a result, it may be directly or indirectly influenced by gut bacteria. The activation of the immune system by gut bacteria may have consequences for *T. cruzi* persistence and transmission, as has been seen in *Anopheles* mosquitoes where presence of gut bacteria induces a strong immune priming effect via hemocyte differentiation following ookinete escape from the midgut, and subsequently reduces the survival of *Plasmodium* in the mosquito [70]. However, our results suggest that within the gut, there is lower activation of the host immune response relative to immune factors in the hemolymph.

The triatomine microbiome has been implicated in diverse interactions with *T. cruzi,* recently reviewed in [86]. Surveys of microbes in kissing bug guts infected with *T. cruzi* describe variable impacts on microbial diversity and abundance [81,87–90]. Tripartite interactions of *T. cruzi*, the host immune system, and microbiome have been documented [15,52,53,91–95], including activation of various host immune effectors by *T. cruzi* which suppress or alter microbial communities in the gut. In *R. prolixus*, infection with *T. cruzi* induces expression of antimicrobial genes which reduces the microbial community [91,93]. In *T. infestans,* infection with *T. cruzi* leads to expression of TiAP, an antimicrobial peptide active against Gram-negative bacteria [92]. Taken together these results indicate that *T. cruzi* manipulates the host immune system and, either directly or indirectly, the microbiome. How these interactions alter immune priming by *R. rhodnii* or how immune priming influences *T. cruzi* infection in *R. prolixus* are important outstanding questions in the field.

Our study provides evidence that the gut microbiome in kissing bugs plays an essential role in activating the host immune system against pathogens in the hemocoel. The nature of the immune priming appears to vary based on the identity of the gut microbe in question, as symbiotic microbes provide a stronger protective effect than non-symbiotic commensals. Both Gram-positive and Gram-negative bacteria were able to activate both the Toll and IMD pathways, which were both essential for immune activation regardless of the bacterial challenge, revealing that our understanding of insect immunity in kissing bugs and possibly other hemipterans, largely based on studies in *Drosophila* and other holometabolous insects, is not complete.

## Materials and methods

### Insect maintenance

*Rhodnius prolixus* were obtained from the lab of Dr. Ellen Dotson at the Centers for Disease Control and Prevention through BEI Resources. Insects were reared at 28 °C with a photoperiod of 12 h of light and 12 h of dark and 80% relative humidity. General colony insects were kept in 1 L Nalgene containers and regularly fed defibrinated rabbit blood (Hemostat Laboratories, Dixon, CA) inoculated with *R. rhodnii* bacteria in the exponential phase of growth through an artificial membrane feeder.

### Generation of axenic and gnotobiotic nymphs

*R. prolixus* eggs were collected 7 days after being laid then placed in a sterile cell collection basket and washed with 70% ethanol for 5 minutes followed by 3 minutes in 10% povidone-iodine solution, then another 5-minute wash in 70% ethanol, followed by three rinses in autoclaved deionized water. Sterilized eggs were then transferred to autoclaved glass containers enclosed in sterile Nalgene containers with gas-exchange tape covering an air hole. Sterility was validated by screening total genomic DNA from insects with PCR to amplify a 16S rDNA gene with the universal primers 27F and 1492R (S1 Table). No bands were observed in axenic nymphs. Gnotobiotic nymphs were generated by feeding axenic first instar nymphs a blood meal inoculated with 10^6^ CFU/mL of *R. rhodnii* *or* *E. coli*. Nymphs were fed at every instar approximately 2 weeks after molting. Gnotobiotic states were confirmed through qPCR on DNA extracted from whole bodies of nymphs using primers specific to the *gyrB* sequence of each bacteria (S1 Table).

### Bacterial strains

*Rhodococcus rhodnii* (NRRL B-16535) was obtained from ATCC and grown at 28 °C in liquid Luria-Broth (LB). *Escherichia coli* MG1655 was a gift of Eric Stabb and was grown in liquid LB at 37 °C. *Micrococcus luteus* NCTC 2665 bacteria was a gift from Michael Strand and was grown in liquid LB at 37 °C. Bacterial titers were determined by measuring the OD_600_ of cultures on a Beckman Coulter DU640 spectrophotometer and then plating out serial dilutions of culture on LB agar plates to correlate OD_600_ with Colony Forming Units (CFU) counts.

### Bacterial immune challenge

Bacterial immune challenge in kissing bugs was performed on either Rpro^Axn^, Rpro^Ec^, or Rpro^Rr^ 4^th^ instar nymphs that were two weeks post molt. Bacteria were injected intrathoracically with 2 μl of 10^8^, 10^6^, or 10^4^ CFU/ml of an overnight culture. For *per os* infection, bugs were given a blood meal inoculated with cells pelleted from 0.5 ml of 10^8^ CFU/ml of an overnight culture. Nymphs were challenged with either *R. rhodnii*, *M. luteus*, *E. coli*, or sterile *Aedes* saline. Bacteria were collected by centrifugation at 8,000 x g for 5 minutes and resuspended in sterile *Aedes* saline. A group of nymphs received a stab wound without injection and a group of nymphs were unaltered and left as a naïve treatment group. Twenty-four hours after injection, whole guts and fat body were dissected out in sterile PBS and stored at -80 °C. For oral infection, 4^th^ instar Rpro^Axn^ or Rpro^Rr^ nymphs that were two weeks post molt were fed a blood meal containing 10^6^ CFU/ml of either *E. coli* or *M. luteus.* Guts and fat body were collected via dissection 24h post-blood meal and tissues were frozen at -80 °C until RNA extraction was performed.

### Real-time quantitative PCR (qPCR)

For analysis of gene expression, axenic and gnotobiotic individuals’ total RNA was isolated from homogenized tissues (gut or fat body) using the Direct-zol 96 RNA MagBead Kit (Zymo Research) and KingFisher Apex extraction system. Total RNA was subject to DNAse treatment with the Turbo DNA-free kit (Thermo Fisher Scientific) according to manufacturer’s instructions. Purified, DNased RNA quantification and purity was validated using a NanoDrop spectrophotometer to measure absorbance ratios. One hundred nanograms of RNA was reverse transcribed using iScript Reverse Transcription Supermix (Bio-Rad). qPCR was performed on synthesized cDNA in quadruplicate using the QuantiNova SYBR Green master mix (Qiagen) in a total volume of 20 µl with 0.5 mM of each primer on a Roche LightCycler96 system or a Qiagen Rotor Gene system. For each gene tested, 4 technical replicates and 5 biological replicates were performed. Absolute quantification of genes was performed as previously described [22] using standard curves of pSCA plasmids containing qPCR products. All qPCR primer pairs had an efficiency of > 0.85.

### Survival analysis

All bacteria were grown in LB media overnight to an OD_600_ = 1. *E. coli*, and *M. luteus* were grown at 37 °C while *R. rhodnii*, was grown at 28 °C. Cells were centrifuged at 6,000 rpm and resuspended in sterile *Aedes* saline to a concentration of 5 x 10^7^ CFU/ml. Fourth instar Rpro^Axn^, Rpro^Rr^, or Rpro^Ec^ nymphs 2 weeks post-molt were injected with 2 µl (10^6^ CFU) of either *E. coli*, *R. rhodnii*, *M. luteus*, or sterile saline with a sterile Hamilton syringe using a Micro4 syringe pump controller (World Precision Instruments). Lower infective doses were tested in Rpro^Axn^ and Rpro^Rr^ by diluting cultues of *E. coli* to 10^2^ CFU and 10^4^ CFU. Nymphs were placed individually into wells of sterile 24-well polystyrene cell culture plates for observation and mortality was observed daily for 21 days post-injection.

### Bacterial clearance in hemolymph

Bacterial abundance in hemolymph was measured by collecting hemolymph from 4^th^ instar nymphs after challenge with kanamycin resistant *E. coli* as described above. To test for the presence of *R. rhodnii* in the hemolymph, hemolymph was collected from Rpro^Rr^ bugs not injected with *E. coli*. All legs were removed with forceps, then an individual was placed inside a sterilized, filtered p1000 pipette tip inserted into a 2 mL microcentrifuge tube, which was then centrifuged at 2000 RCF for 10 min at 15 °C, resulting in the collection of 2–3 µl of hemolymph from an individual. Hemolymph of 4 individuals per treatment was pooled, diluted in 20 µl of sterile PBS, and spread on LB agar plates with 50 µg/ml of kanamycin sulfate, then incubated for 24 h at 37 °C and the number of CFUs were counted. For each treatment, three biological replicates were performed.

### Quantification of antimicrobial hemolymph activity

Antimicrobial activity of kissing bug hemolymph was measured using a zone of inhibition assay as described by [26,27]. A culture of *M. luteus* was grown overnight in LB at 37 °C, then 1 mL of culture was added to 10 mL of sterile, cooled liquid LB agar. The *M. luteus*- LB agar solution was mixed and poured into Petri plates. After solidifying, 1 mm diameter holes were created in the agar using a sterile glass Pasteur pipette. Hemolymph was collected from individual nymphs as described above, and 1 µl of hemolymph was placed in each hole and the plates were incubated overnight at 37 °C. The diameters of the individual zones of bacterial growth inhibited were measured using an ocular micrometer on a stereo dissecting microscope. Two independent trials were conducted, and each trial consisted of 10 bugs per treatment group.

### Antibiotic clearing/recolonization of *R. rhodnii*

To investigate the impacts on survival of microbiome recolonization, we inoculated 3^rd^ instar Rpro^Axn^ bugs with *R. rhodnii* (Rpro^Axn + Rr^) through a blood meal as previously described. Bugs were allowed to develop to the 4^th^ instar when the presence of *R. rhodnii* was confirmed in a subset of bugs via qPCR. Two weeks after molting bugs were immune challenged with *E. coli* and survival was monitored as previously described. To determine the effects of removal of *R. rhodnii* on host immune function, 3^rd^ instar Rpro^Rr^ were fed a bloodmeal containing either 150 µg/ml of kanamycin or a bloodmeal containing 150 µg/ml of kanamycin along with kanamycin resistant *R. rhodnii* (Rpro^Rr ^+ Kan and Rpro^Rr-KanR ^+ Kan). Bugs were confirmed to be removed of *R. rhodnii* or confirmed to still harbor *R. rhodnii* via qPCR as described above. All bugs were then allowed to molt to the 4^th^ instar then immune challenged as previously described and survival was monitored.

### RNAi-mediated immune suppression

PCR Primers containing the minimal T7 promoter sequence were designed to amplify 400–500 bp of *relish, dorsal, or egfp* (S1 Table). Total RNA was extracted from the fat bodies of 4^th^ instar nymphs, DNased, and reverse transcribed as described above. The PCR product was subsequently cloned to the pSCA vector using the Strataclone PCR cloning kit (Agilent). Target DNA was amplified by PCR from isolated plasmid DNA. dsRNA was synthesized using the MEGAscript RNAi kit (ThermoFisher Scientific) according to the manufacturer’s instructions. Synthesized dsRNA was precipitated with sodium acetate and ethanol, then resuspended to 2 μg/μl in *Aedes* saline. Fourth instar nymphs were injected with 1 μl of dsRNA, then allowed to recover for 1 week before immune challenge as described previously. Knockdown of *relish* or *dorsal* was confirmed by qPCR on cDNA extracted from treated nymphs as described previously.

### Hemocyte quantification and inactivation

Hemolymph was collected from 4^th^ instar nymphs that were 2 weeks post-molt. Hemolymph was collected via perfusion of 100 µL of cold *Aedes* saline injected through the abdomen via insulin syringe. The samples were stored on ice until hemocyte numbers were counted using a Neubauer hemocytometer and an inverted stereo microscope. Two counts per insect were conducted and the average of the two counts was used for each of 5 individuals per treatment.

To reduce hemocyte activity, fluorescent latex microbeads (Polyscience, Fluoresbrite Microspheres 2.00 µm) were diluted to 10^8^ beads/μl in *Aedes* saline and injected into the hemolymph of 22 4^th^ instar nymphs that were two weeks post-molt. Beads were confirmed to be engulfed by host hemocytes by observation with an epifluorescence microscope (Leica). Four hours after injection with beads, nymphs were challenged with injection of either *E. coli* or sterile *Aedes* saline then monitored for survival as previously described.

### DOPA conversion assay

Hemolymph was collected as described above from Rpro^Axn^, Rpro^Ec^, or Rpro^Rr^ injected with either *E. coli* or *M. luteus*. DOPA conversion was measured as described in [35]. Briefly, 100 µl of perfused hemolymph was suspended into 100 µl of PBS containing 4 mg/ml DOPA, and added to the wells of a sterile 96 well plate. The plate was then incubated at 28 °C for 1 h in a humidified chamber and absorbance was read at 470 nm and on a µQuant plate reader (BioTek), then returned to the chamber and measured again at 24h. Prior to analysis, background absorbance of blank wells was subtracted from the values of test wells. Four to six bugs per treatment were tested.

### Statistical analyses

Statistical analysis of insect survival was determined using a Cox-proportional hazards model followed by a log-rank test for pairwise comparisons via R package *survminer* and *survival*. Bacterial clearance was analyzed using a Wilcoxon rank-sum test with a Benjamini-Hochberg correction for multiple comparisons. Statistical analysis of gene expression and hemocyte counts was performed using an aligned-rank transformed ANOVA test followed by a Tukey post-hoc test using the R package *ARTool* [96]. DOPA conversion assay data was analyzed via ANOVA and Tukey post-hoc tests. Data files and R scripts used in the study are found in the supplemental online data.

## Supporting information

S1 Fig Survival of axenic and gnotobiotic insects challenged with different pathogen doses. (EPS)

S2 Fig Zone of inhibition assays of naïve insects. (EPS)

S3 Fig Titer of bacteria in antibiotic-treated insects. (EPS)

S4 Fig qPCR confirmation of *dorsal* and *relish* RNAi knockdown. (EPS)

S1 Table Primers used in this study. (XLSX)

S2 Table Statistical details of qPCR expression experiments. (XLSX)

S1 Data Zipped folder of R scripts and datafiles used in the manuscript. (ZIP)
